# A new lapsiine jumping spider from North America, with a review of Simon’s *Lapsias* species (Araneae, Salticidae, Spartaeinae)

**DOI:** 10.3897/zookeys.891.38563

**Published:** 2019-11-21

**Authors:** Wayne P. Maddison

**Affiliations:** 1 Departments of Zoology and Botany and Beaty Biodiversity Museum, University of British Columbia, 6270 University Boulevard, Vancouver, British Columbia, V6T 1Z4, Canada University of British Columbia Vancouver Canada

**Keywords:** Guatemala, jumping spider, Lapsiini, México, Venezuela

## Abstract

A new spider genus and species from México and Guatemala, *Amilaps mayana***gen. et sp. nov.**, is described, distinct from other members of the jumping spider tribe Lapsiini (subfamily Spartaeinae) by its four retromarginal cheliceral teeth and the large sclerite cradling the embolus. It is the first living lapsiine known outside of South America. This tribe has received attention recently for new species and genera in Ecuador and Brazil, but Simon’s original four species of *Lapsias*, described from Venezuela in 1900 and 1901, remain relatively poorly known. Accordingly, new illustrations of Simon’s type material are given, and a lectotype is designated for *L. cyrboides* Simon, 1900. The three forms of females in Simon’s material from Colonia Tovar, Aragua, are reviewed and illustrated, and they are a tentatively matched with the three male lectotypes of his species from the same location.

## Introduction

For more than 100 years after Eugene Simon’s (1900) description of the jumping spider genus *Lapsias* Simon, 1900, the only known species were the four he described from Venezuela (Simon, 1900, 1901). Indeed, these were the only species described of the broader group now recognized as the Lapsiini, one of only two salticid groups in the New World that fall outside the major subfamily Salticinae (the other being the Lyssomaninae). Considerably more lapsiine diversity has been revealed since 2006 through work by Maddison (2006, 2012), Makhan (2007), Ruiz and Maddison (2012), and Ruiz (2013), giving us now five described genera containing 21 species (WSC 2019). All of the living lapsiine species known to date are from South America, but recently García-Villafuerte (2018) described a fossil of *Galianora* Maddison, 2006 from Miocene amber in Chiapas, México.

Here I report the north-westernmost known living lapsiine, *Amilaps mayana* sp. nov., from southern México and Guatemala. In addition, new illustrations of Simon’s four species of *Lapsias* from Venezuela are provided to supplement Galiano’s (1963) redescriptions, and the matching of males and females is reconsidered.

## Materials and methods

The preserved specimens were examined under both dissecting microscopes and a compound microscope with reflected light. Photographs were taken under an Olympus SZ61 stereo microscope (bodies) and a Nikon ME600L compound microscope (palpi) and focus stacked using Helicon Focus 4.2.7. Drawings were made with a drawing tube on an Olympus BH-2 compound microscope (*Amilaps mayana* sp. nov.) and a Nikon ME600L compound microscope (Simon’s species).

Terminology is standard for Araneae. Measurements are given in millimetres. Carapace length was measured from the base of the anterior median eyes not including the lenses to the rear margin of the carapace medially; abdomen length to the end of the anal tubercle.

### Abbreviations

**AME** anterior median eyes;

**ALE** anterior lateral eyes;

**PME** posterior median eyes;

**PLE** posterior lateral eyes;

**RTA** retrolateral tibial apophysis.

### Museum abbreviations

**MCZ**Museum of Comparative Zoology, Harvard University (G. Giribet);

**AMNH**American Museum of Natural History, New York (L. Prendini);

**MNHN**Muséum national d’Histoire naturelle, Paris (C. Rollard).

## Taxonomy

### Amilaps gen. nov.

Taxon classificationAnimaliaAraneaeSalticidae

DD7E7ECD-5F36-5C25-B368-B53823BBF6BE

http://zoobank.org/AEE550A1-9490-41C9-8D0E-EAF544D338F0

#### Type species.

*Amilaps mayana* sp. nov.

#### Etymology.

An arbitrary combination of letters, composed to contain a reference to the Mayan word for spider (“äm”, Christensen 1987) and to *Lapsias*, to be treated grammatically as feminine.

#### Diagnosis.

Differs from all described lapsiines in having a large sclerite (**p** in Fig. 3) cradling the tip of the embolus, and in having four retromarginal teeth on the chelicerae (two in all others; see Ruiz and Maddison 2012, Maddison 2012, Ruiz 2013). Differs from *Lapsias*, *Soesiladeepakius*, and *Thrandina* in lacking a prolateral pre-embolic spermophore loop (see Ruiz and Maddison 2012), although the loop may be present on the retrolateral side (see below under “Relationships”). Unlike most *Lapsias* species, *Amilaps* has the PME displaced medially, as far as the medial edge of the ALE.

#### Relationships.

The four retromarginal cheliceral teeth suggest that *Amilaps* is outside a clade including all previously described lapsiine genera, which share the synapomorphy of a reduction to two teeth (Ruiz and Maddison 2012) from the plurident condition in other Spartaeinae. There are no clear characters linking *Amilaps* to any particular lapsiines: it lacks the highly reduced RTA of *Lapsias*, the round tegulum of *Galianora*, the large PME and robust median apophysis of *Thrandina*, and the many peculiarities of *Soesiladeepakius* and *Lapsamita*. The spermophore of *Amilaps* appears to lack the pre-embolic loop approaching the median apophysis, widespread in lapsiines (e.g., Figs 14, 21, 30, 41; Maddison 2012: figs 7, 11, 12; see Ruiz and Maddison 2012: character 17). In *Amilaps mayana* the spermophore does in fact closely approach the median apophysis (MA), but on the retrolateral side of the bulb. In ventral view, it passes just retrolateral to the MA, but in retrolateral view it can be seen to be curved, reaching its ventralmost point just proximal to the MA. If this is the same pre-embolic loop but displaced retrolaterally, it hints to the possibility that the base of the embolus of *A. mayana* may be unusually large, occupying a large proportion of the prolateral side of the bulb.

If *Amilaps* is outside the clade of previous lapsiines, then an open question is whether it belongs with them at all. The tribe Lapsiini has no known morphological synapomorphies (Maddison 2015) other than the reduction in cheliceral teeth (Ruiz and Maddison 2012). Our understanding of morphology gives little reason to expect that salticids in the Americas left over once salticines and lyssomanines are removed would form a clade, but the molecular data suggests this, at least among those species studied (Maddison et al. 2014). *Amilaps* is exactly that: a generalized salticid that is not a salticine or lyssomanine. Were it to have been found in New Guinea, *Amilaps* would fit equally happily among the cocalodines according to our current knowledge. Thus, its current placement among the lapsiines is tentative.

### Amilaps mayana sp. nov.

Taxon classificationAnimaliaAraneaeSalticidae

BE0AD9A7-DDE0-59C1-BE9A-61DA36B64B5A

http://zoobank.org/154D15D5-3292-4465-B6C6-11768434EDD6

Figs 1–11

#### Type material.

Holotype in MCZ: male, with label “MCZ, MEXICO: TABASCO: 2.4 km E of Teapa, Grutas de Cocona, ca. 17°33'N, 92°56'W 7 July 1983 W.Maddison 83-089 forested steep slope, ca. 250 ft. el.”. The recorded latitude is likely incorrect; the specimen was collected near the entrance to the Grutas, which is at ca. 17.564N, 92.929W.

#### Etymology.

Refers to the distribution of this species in the lands of the Maya.

#### Description.

**Figures 1–11.**
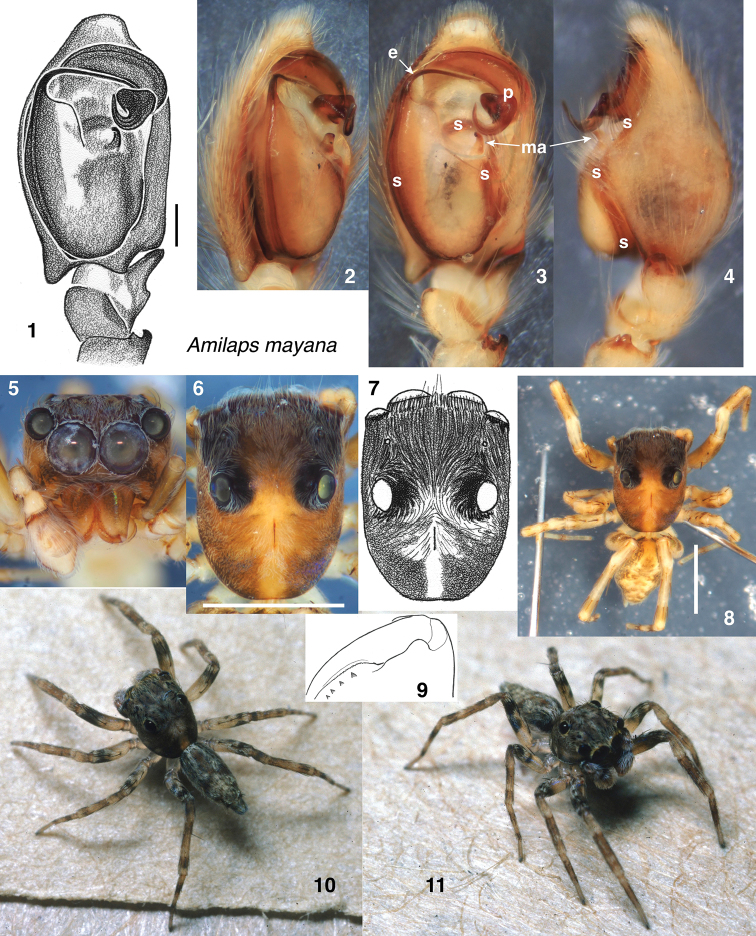
*Amilaps mayana*, sp. nov, male holotype **1–4** palp **1** ventral view **2** prolateral view **3** ventral view **4** retrolateral view **5** face **6, 7** carapace, dorsal view **8** body in alcohol **9** posterior ventral view of chelicera showing four retromarginal teeth **10, 11** living specimen. Abbreviations: **e** embolus, **s** spermophore, **ma** median apophysis, **p** sclerotized projection serving as conductor. Scale bars: 0.1 mm (**1**); 1.0 mm(**6, 8**).

**Male** (holotype). Carapace length 2.0; abdomen length 1.7. ***Carapace*** (Figs 6, 7) with long fovea; anterior eye row approx. as wide as carapace, and wider than posterior row. PME small, displaced medially to lie behind medial edge of ALE. Ocular area medium brown under alcohol and darker around eyes, dusted with dull brown and tan scales that are oriented concentrically around the unusually large PLE. Thoracic area brown, with paler medial longitudinal band, and paler spots just above each of the leg coxae. ***Clypeus*** (Fig. 5) narrow and with a few scattered whitish hairs and scales. ***Chelicerae*** vertical and relatively small. Four small but distinct teeth on retromargin of chelicerae (Fig. 9); promargin not observed (on the specimen from Guatemala, three promarginal teeth). ***Palp*** (Figs 1–4) with embolus arising on prolateral side, narrowing abruptly, then bending directly to the retrolateral, where it meets a large sclerotized projection (**p** in Fig. 3) that envelops it so completely that the terminal third of the embolus is most easily seen as a dark line within the projection; the tip of the embolus rests within the tip of the projection. The projection consists of a plate at the distal edge of the bulb, which then narrows before swelling and curving to a point that projects ventrally. (Regarding its homology to the conductor in *Lapsias*, see comments below.) Median apophysis distinct (separated from the tegulum by a membrane) but relatively small, almost hidden by the sclerotized projection. Cymbium with proximal prolateral conical projection. Retrolateral tibial apophysis a short flange (Fig. 4) whose ventral edge extends proximally and forms a round pocket facing retrolateral side. Patella with two retrolateral apophyses, the larger one being hooked. ***Legs*** (Figs 10, 11) pale honey-coloured, darkening to nearly black on distal half of femora, and with broad darker annuli on tibiae and metatarsi. First tibia macrosetae as follows: three pairs of ventral, two to three anterior lateral, two posterior lateral, and one dorsal. First metatarsus macrosetae as follows: two ventral pairs, two anterior lateral, and two posterior lateral. Fourth legs distinctly longest; leg formula 4132. ***Abdomen*** (Figs 8, 10) brown above, with paler undulating medial longitudinal pale band.

#### Additional material.

Male in AMNH from Guatemala: Petén: Cueva de Olla, Poptún. 8 April 1989. A. Cobb. The specimen is missing legs and is mostly disarticulated, but its structure including the distinctive palp matches the holotype.

#### Data for material examined.

México • 1 ♂, holotype; Tabasco, 2.4 km E of Teapa, Grutas de Cocona; 17.564N, 92.929W; 7 Jul. 1983; W. Maddison leg.; collecting event WPM#83-089; MCZ. Guatemala • 1 ♂; Petén, Poptún, Cueva de Olla; 8 Apr. 1989; A. Cobb, leg.; AMNH.

#### Natural history.

My field notes for the holotype indicate it was found on a limestone rock face, and the back of the vial’s label says “on limestone cliff face on forested slope”. Both the holotype from México and the male from Guatemala (according to its locality) were associated with caves. The holotype was not in the cave, but on a cliff near the cave.

### Lapsias

Taxon classificationAnimaliaAraneaeSalticidae

Simon, 1900

14982982-431D-598B-A7F8-D3EA8ED7C40F

#### Type species.

*Lapsias estebanensis* Simon, 1900, by original designation.

Although Galiano (1963) redescribed Simon’s original four species, her illustrations are limited in number and detail. Thus, I give new figures of Simon’s original four species, including the first published figures of their bodies and more detailed illustrations of their genitalia. Among Simon’s specimens are three forms of female, only one of which (under *L. cyrboides* Simon, 1900) was described by Simon and Galiano. As these females are all from the same site (Colonia Tovar) from which the males of three *Lapsias* species were described, we are faced with a puzzle as to which females match which males. This is considered below under the notes for each species.

All four of Simon’s species have two retromarginal teeth on the chelicera, and three pairs of ventral macrosetae on both the tibia and metatarsus of leg 1. The median apophysis of the palp is a long narrow blade, hooked at the tip and separated from the tegulum by a membrane. There is a small apophysis just retrolateral from the base of the embolus in *L. estebanensis*, *L. tovarensis* Simon, 1901, and possibly *L. ciliatus* Simon, 1900 (see **c**? in Figs 14, 43) that by position is likely homologous to that called the conductor by Maddison (2012) in *L. canandea* Maddison, 2012 and in *Thrandina* species (see discussion by Ruiz 2013). The sclerite functioning as a conductor in *Amilaps mayana* (**p** in Fig. 3) is likely not homologous, given its more distal position outside the loop of the spermophore. The female spermathecae of all three species (Figs 28, 38, 49) are thick-walled and bear a pale rough-edged extension to the anterior (most easily seen in Fig. 28; partially hidden behind the fertilization ducts in Figs 38 and 49).

### Lapsias estebanensis

Taxon classificationAnimaliaAraneaeSalticidae

Simon, 1900

EC6F224C-93F1-5709-BF70-A309A791C64D

Figs 12–18

#### Type material.

In MNHN, 2 males from La Cumbre, San Esteban, Carabobo State, Venezuela, with label “21196 Laps. estebanensis E.S., S. Esteban! La Cumbre!”. Galiano (1963) designated one male as lectotype, which I presume to be that in a separate microvial with her label “Typus? M.E. Galiano II 1959”. The type vial also has a recent label “det Szűts 0015”. Because the old handwritten label was fragile and fragmenting, I made a copy, which I added to the type vial.

#### Notes.

This is the most robust of the four Venezuelan species, with males having enlarged chelicerae (Figs 15–17). The retromarginal tooth closest the fang is larger and curved (Fig. 18). The palp bears a close resemblance to that of *L. tovarensis*, but differs in the shorter, straighter embolus and distinctly larger apophysis (**c**? in Fig. 14) accompanying the embolus.

**Figures 12–18.**
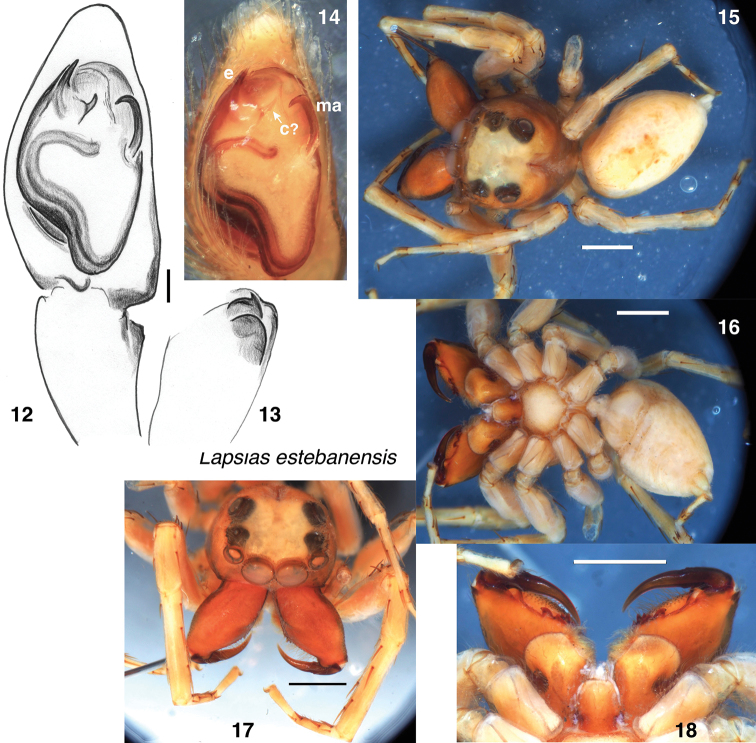
*Lapsias estebanensis*, lectotype male **12–14** palp **12** ventral view **13** retrolateral view of tibia **14** ventral view **15** dorsal view of body **16** ventral view of body **17** oblique view of prosoma **18** chelicerae from below. Abbreviations: **e** embolus, **c**? scerite homologous to that called the conductor in other lapsiines, **ma** median apophysis. Scale bars: 0.1 mm (**12**); 1.0 mm (otherwise).

### Lapsias ciliatus

Taxon classificationAnimaliaAraneaeSalticidae

Simon, 1900

BBA24748-C88B-5FA0-90E2-B96FC78275BD

Figs 19–29

#### Type material.

In MNHN Paris, 25 males from Colonia Tovar, Aragua State, Venezuela, most in a single vial with label “21083 Laps. ciliatus E.S., Tovar!” and more recent label “det Szűts 0012”. When I received the specimens from the MNHN, one male matching this species was in a separate vial without label except one in Galiano’s handwriting reading “Typus? M.E. Galiano II 1959” and another “det Szűts 0013”. Insofar as Galiano (1963) indicated she designated a lectotype from the type vial, this specimen can be safely considered that specimen. I have therefore made a copy of the label “21083 Laps. ciliatus E.S., Tovar!” and placed it in that male lectotype’s vial. The same applies to a female separated and with only Galiano’s label “Allotypus ♀ det. M.E. Galiano II 1959”. The vial with most specimens also includes 7 females, which cannot be considered type material because Simon’s description makes no mention of females.

#### Notes.

The female is illustrated for the first time in Figs 27–29. The epigynal openings are beneath a common central hood. Although Galiano separated off a female and labelled it as allotype, neither she nor Simon gave any acknowledgement or description of a female of *L. ciliatus*. The matching of these females to males of *L. ciliatus* is reasonably secure, even though three species of *Lapsias* occur at Colonia Tovar. The females of form shown in Figs 27–29 and the males matching the lectotype appear to have been abundant together, judging by the numbers of specimens. Both are larger and more robust, with wider carapaces, than the other two smaller, more delicate species from Colonia Tovar (*L. cyrboides* and *L. tovarensis*). Both male and female show a faint pale spot just posterior to the PLE.

**Figures 19–29.**
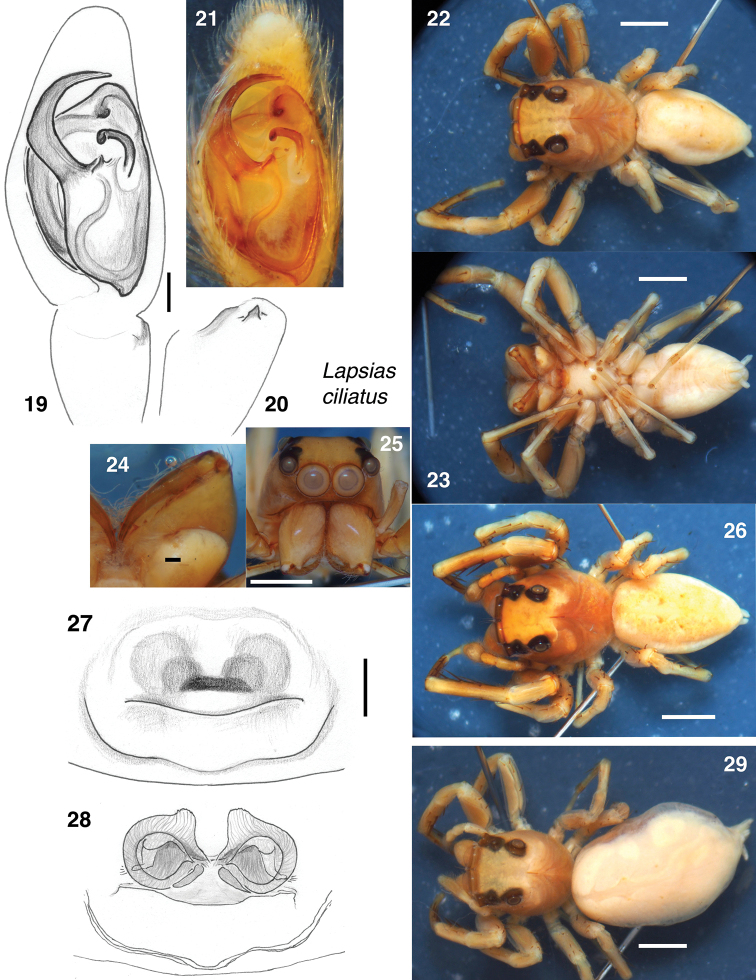
*Lapsias ciliatus*, lectotype male (**19–25**) paralectotype (**26**), and associated female (**27–29**). **19–21** Male palp **19** ventral view **20** retrolateral view of tibia **21** ventral view **22** male body from above **23** body from below **24** chelicera from below **25** face **26** paralectotype male body from above **27** epigyne from below **28** vulva from above **29** body of same female as **27, 28**. Scale bars: 0.1 mm (**19, 27**); 1.0 mm (otherwise).

### Lapsias cyrboides

Taxon classificationAnimaliaAraneaeSalticidae

Simon, 1900

658ED9F6-309A-5704-B883-EA9244F5743E

Figs 30–40

#### Type material.

In MNHN, 3 males, 4 females, 3 juveniles from Colonia Tovar, Aragua State, Venezuela, in a vial with label “20924 Laps. cyrboides E.S., Tovar!” and a more recent label “det Szűts 0014”. Galiano (1963) designated one male as a lectotype, in separate microvial with her label “Typus? M.E. Galiano II 1959”. She mentions one female designated also as lectotype, but no female is separated and labelled as such. Because Galiano (incorrectly) designated two lectotypes, the name is not yet fixed to a single specimen. This ambiguity is resolved by designating her male lectotype as the only lectotype.

#### Notes.

Simon (1900) described a male and female. However, as noted by Galiano (1963), there are two species of female among the four females in the type vial, similar in body but easily distinguished by the epigyne. Two of the females (Figs 37–40) have an anteriorly placed guide (Fig. 37), while the other two females (Figs 48–51) lack such a guide and instead show two wing-shaped atria extending laterally (Fig. 48). It is reasonable to assume that these two kinds of female belong to the two smaller-bodied *Lapsias* at Colonia Tovar, *L. cyrboides* and *L. tovarensis*. Under *L. cyrboides* Simon described the female kind with anterior guide (“Plaga genitalis...longior quam latior”), but he did not justify this choice nor even mention the second form of female. Galiano followed Simon’s choice of matching female. The two forms of female are approximately the same size and carapace shape and are too faded to supply distinctive markings by which to match to the males. Nonetheless, I tentatively support Simon’s and Galiano’s matching based on an expected correlation between the form of the female’s guide and that of the male’s RTA. An anterior guide is expected to be associated with an extraordinary RTA, for instance, as in *Papuamyr omhifosa* Maddison & Szűts, 2019 (Maddison and Szűts 2019). Males of *L. ciliatus* and *L. tovarensis* have small RTAs, suggesting a simple or small guide along the epigastric furrow, as is common in salticids, while the male of *L. cyrboides* has an unusual dorsally projecting tibial apophysis, which predicts an unusually-placed female guide. Thus, the female with anterior guide is tentatively considered that of *L. cyrboides*, and the female with wing-shaped atria is considered that of *L. tovarensis*.

**Figures 30–40.**
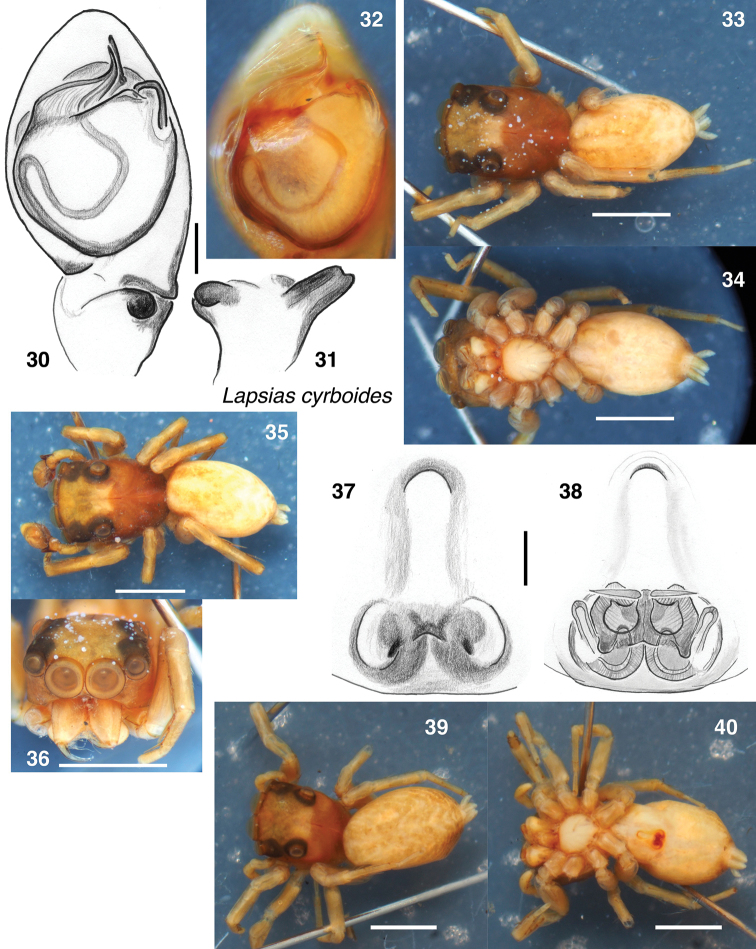
*Lapsias cyrboides* lectotype male (**30–34, 36**), paralectotype male (**35**), and female tentatively considered of this species (**37–40**). **30–32** Palp **30** ventral view **31** retrolateral view of tibia **32** ventral view **33** body from above **34** body from below **35** paralectotype male body from above **36** lectotype male face **37** epigyne from below **38** vulva from above **39** female body from above **40** body from below. Scale bars: 0.1 mm (**30**); 1.0 mm (otherwise).

### Lapsias tovarensis

Taxon classificationAnimaliaAraneaeSalticidae

Simon, 1901

740B85B4-48D9-5E5B-8009-6F638A7DCE5A

Figs 41–51

#### Type material.

In MNHN, three males from Colonia Tovar, Aragua State, Venezuela, with label “21092 Laps. tovarensis E.S., Tovar!”. Galiano (1963) designated one male as lectotype, in separate microvial with her label “Typus? M.E. Galiano II 1959”.

#### Notes.

This is one of the two smaller-bodied species from Colonia Tovar. See the discussion under *L. estebanensis* for how to distinguish it from that species, and the discussion under *L. cyrboides* regarding the identity of the female.

**Figures 41–51.**
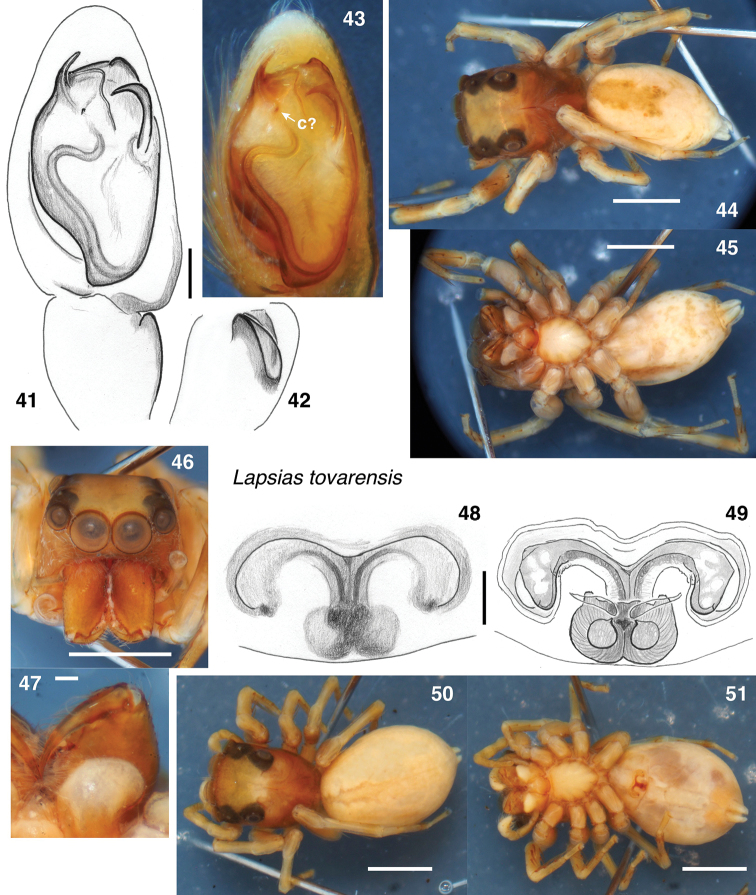
*Lapsias tovarensis* lectotype male (**41–47**) and female tentatively considered of this species (**48–51**). **41–43** Palp **41** ventral view **42** retrolateral view of tibia **43** ventral view **44** body from above **45** body from below **46** face **47** chelicerae from below **48** epigyne from below **49** vulva from above **50** female body from above **51** body from below. Abbreviations: **c**? sclerite homologous to that called the conductor in other lapsiines. Scale bars: 0.1 mm (**41**); 1.0 mm (otherwise).

## Supplementary Material

XML Treatment for Amilaps

XML Treatment for Amilaps mayana

XML Treatment for Lapsias

XML Treatment for Lapsias estebanensis

XML Treatment for Lapsias ciliatus

XML Treatment for Lapsias cyrboides

XML Treatment for Lapsias tovarensis
